# Anti-Obesity Effect of *Bombus ignitus* Queen Glycosaminoglycans in Rats on a High-Fat Diet

**DOI:** 10.3390/ijms18030681

**Published:** 2017-03-22

**Authors:** Mi Young Ahn, Ban Ji Kim, Ha Jeong Kim, Hyung Joo Yoon, Sang Duck Jee, Jae Sam Hwang, Kun-Koo Park

**Affiliations:** 1 Department of Agricultural Biology, National Academy of Agricultural Science, Rural Development Administration (RDA), Wanju-Gun 55365, Korea; kimbj0826@naver.com (B.J.K.); musical2058@daum.net (H.J.K.); Yoonhj@korea.kr (H.J.Y.); sdji11@korea.kr (S.D.J.); jshwang@korea.kr (J.S.H.); 2 Pharmacogenechips Inc., Chuncheon 200-160, Korea; pkk0526@naver.com

**Keywords:** anti-obesity effect, glycosaminoglycan from bumblebee (queen of *Bumbus ignitus*), high fat dieted rat

## Abstract

The mechanism of functional insect glycosaminoglycan (GAG) on obesity caused a high fat diet has not yet been elucidated. Therefore, insect glycosaminoglycans derived from *Isaria sinclairii*, *Bombus ignitus* (a type of bumblebee) queen, and *Gryllus bimaculatus* were purified and investigated as a potential functional food. 14-week old male Wistar rats were fed a high-fat diet (HFD) for 6 weeks. There were five groups that received daily intraperitoneal administration of phosphate buffered saline (PBS, control), GbG (GAG from *Gryllus bimaculatus*) 10 mg/kg, ISG (GAG from *Isaria sinclairii*) 10 mg/kg, IQG (GAG from *Bombus ignites*) 10 mg/kg, or Pravastatin (2 mg/kg). All treatments were performed for one month. IQG produced a potential anti-inflammatory effect with the inhibition of c-reactive protein and sero-biochemical parameters of phospholipids and free fatty acids indicative of an anti-hyperlipidemic effect. Abdominal and epididymidal fat weight were reduced in conjunction with a mild increase in body weight. The level of laminin in HMVEC-C cells or fibronectin in HFD rat hepatocytes was significantly affected by these GAG treatments, which regulated adipogenesis and adipocyte function. Compared to the control rats, IQG-treated rats displayed up-regulation of 87 genes (test:control ratio >2.0) including fatty acid synthase and 3-hydroxy-3-methylglutaryl-coenzyme A reductase, with the down-regulation of 47 genes including the uridine diphosphate (UDP) glycosyltransferase 2 families, polypeptidase B, and insulin-like growth factor binding protein 1. The data suggest that IQG could potentially prevent or treat fatty liver or hyperlipidemia.

## 1. Introduction

Glycosaminoglycans (GAGs), including heparin-like polymer, that are derived from vertebrates have diverse pharmacological activities including anticoagulant, antithrombotic, and anti-inflammatory activities [1]. GAGs isolated and purified from various vertebrate and invertebrate tissues have been shown to comprise of an anion sugar sulfate with functional properties [2]. GAGs derived from insects have been studied such as a mosquito heparan sulfate [3], *Isaria sinclairii* GAG capable of reducing blood pressure [4], and *Gryllus bimaculatus* GAG having anti-inflammatory activity [5]. The extracellular matrix (ECM) has been shown to regulate the development and function of numerous tissues and organs. However, there is little understanding of its function in adipose tissue and its matrix proteins: laminin, fibronectin, glycosaminoglycan, etc. [6]. Laminin α4, a specialized extracellular matrix protein surrounding adipocytes, modulates cellular behavior in adipose tissue expansion [6]. As another ECM glycoprotein involved in both physiological and pathological processes, fibronectins are adhesive glycoproteins that exist in tissue matrices and circulate in various fluids of the body [7]. The tripeptide Arg-Gly-Asp (RGD) site is a heparin-binding domain of fibronectin [8]. Through this association, circulating fibronectin modulates blood vessel formation and tumor growth by modifying the amount of and the response to vascular endothelial growth factor (VEGF) [9]. The mechanism of anti-obesity on these insect glycosaminoglycans at the ECM molecular level was studied by DNA microarray investigation. In the previous study, because *G. bimaculatus* (Gb) glycosaminoglycan was recently demonstrated as capable of inhibiting adipose tissue accumulation in rats fed a high-fat diet (HFD) [10], in this experiment, Gb GAG was the positive control. For parameter levels, the weight and fatty acid composition of abdominal fat and epididymidal fat, total cholesterol, low-density lipoprotein-cholesterol, and triglyceride in rats treated with sample GAG, *I. sinclarii* GAG, or *B. ignitus* queen GAG were investigated and compared to those of the negative (PBS) or positive (*G. bimaculatus* GAG or Pravastatin) control group. For the hyperlipidemia and obese rat model, HFD can cause oxidative stress due to lipid peroxidation those results from increased malondialdehyde and protein carbonyl content. In a previous study, anti-obesity effects in obese (fa/fa) Zücker rats and anti-diabetic effects in C57BL/6 obese (ob/ob) mice of *Isaria sinclairii* (Cicada Dongchunghacho, a fungus cultured on silkworm) powder have been documented [11,12]. *I. sinclairii* GAG was designed and proposed with a safer and more effective activity than *I. sinclairii* as a purified substance, in the glycosaminoglycan form in a HFD rat model.

Another new insect source of GAG is the bumblebee (*Bombus igitus*). *B. ignitus* is used globally for pollination and the medicinal and nutritional uses of other hive products, especially from the queen, have been indicated [13]. We endeavored to make a safe and effective *B. ignitus* queen GAG (designated IQG), and tested its anti-atherosclelosis activity by determining nitric oxide (NO) production in endothelial cells and antithrombotic activity in vitro; furthermore, various insect GAGs were applied to the high fat diet rat model experiment in vivo.

As a commercial antilipidemic agent, this study included Pravastatin, a hydroxymethylglutaryl-CoA reductase inhibitor with lipid-lowering activity that has made it popular in the treatment and prevention of atherosclerotic diseases [14]. The antilipidemic activity of IQG was compared with GAG from Gb (GbG), *I. sinclarii* GAG (ISG), and Pravastain as positive controls in a HFD Wistar rat model using sero-biochemical, anti-oxidative and DNA micro array examinations.

Throughout these results, we could demonstrate the potential efficacy of IQG as an anti-lipidemic treatment with anti-obesity; IQG may have potential as a protective nutraceutical for atherosclerosis disorders, including circulatory disorders.

## 2. Results

### 2.1. Body Weight and Adipose Fat Weight Changes

**Figure 1:**
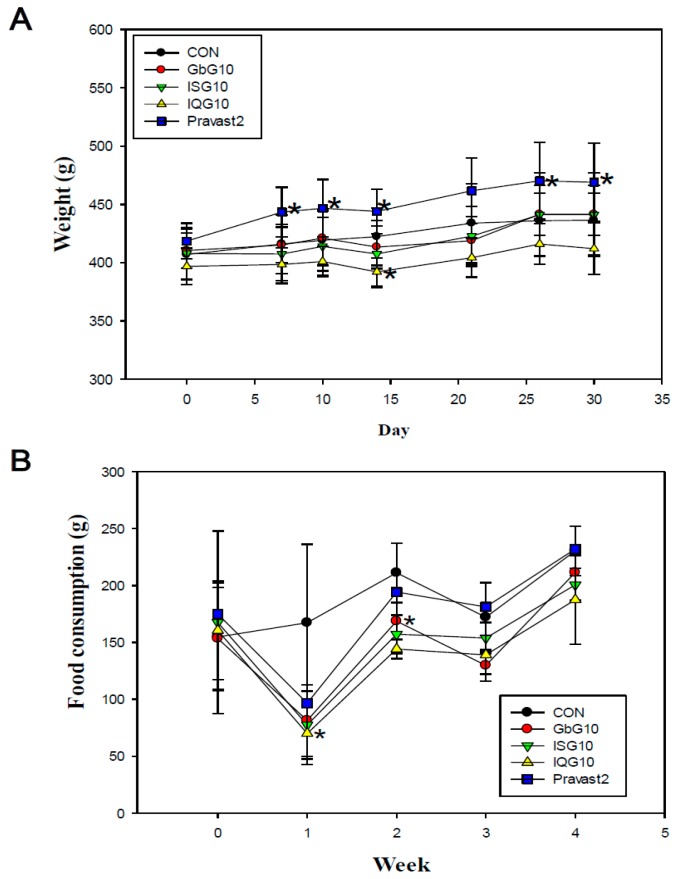
(**A**) Effect of IQG: *B. ignitus* glycosaminoglycan on body weight in high fat (HFD, 60%) diet rats over one month. GbG10: *G. bimaculatus* glycosaminoglycan 10 mg/kg. ISG10: *I. sinclairii* glycosaminoglycan 10 mg/kg. IQG10: *B. ignitus* glycosaminoglycan 10 mg/kg. Pravast2: Pravastain 2 mg/kg. * *p* < 0.05, compared with the control (HFD only) group. (**B**) Food consumption changes in rats treated with IQG on a high fat diet. * *p* < 0.05, compared with control (HFD only) group.

**Figure 2:**
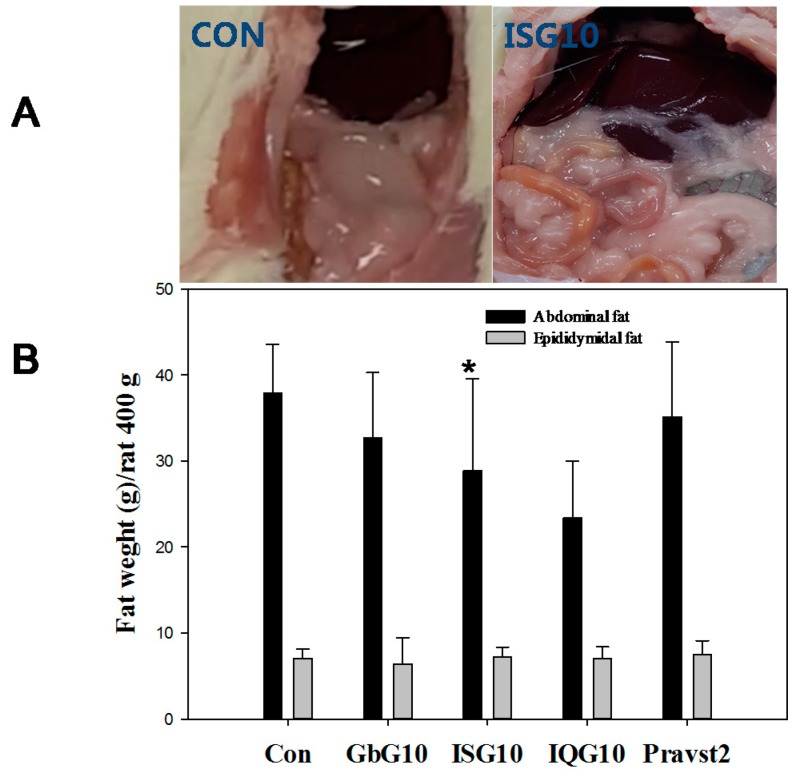
(**A**) Abdominal fat in a Wistar HFD rat. Abdominal fat is shown in the CON (control group) and ISG (ISG10: *I. sinclairii* glycosaminoglycan 10 mg/kg) groups. (**B**) Effect of IQG or ISG on abdominal and epididymidal fat weight on a high fat diet. * *p* < 0.05, compared with the control (HFD only) group. GbG10: *G. bimaculatus* glycosaminoglycan 10 mg/kg. *B. ignitus* glycosaminoglycan 10 mg/kg. Pravast2: Pravastain 2 mg/kg.

There were significant differences in mean body weight between all of the treatment groups during the one month treatment (Figure 1A). The body weight gain of rats treated with IQG or ISG was at a lower level compared to the control. However, the body weight of the Pravastatin group, as an anti-atherosclerosis agent but not as an anti-obesity drug, is higher than that of the control rats. Mean weekly body weight and food consumption are presented in Figure 1A,B, respectively. Mean abdominal fat was also significantly decreased compared to the control (31.29 g for control (CON); 34.49 g for GbG10; 29.50 g for ISG10 (*p* < 0.05 vs. CON); 23.25 g for IQG10; 38.85 g for Pravast2) (Figure 2B). Epididymal fat was not significantly decreased compared to the control (Figure 2B).

### 2.2. Blood Pressure and Heart Rate Changes

Marginal statistically significant differences in blood pressure and heart rate were observed between the 10 mg/kg GbG-, ISG-, and IQG-treated groups compared to the control (Table 1).

### 2.3. Serum Biochemistry

**Figure 3:**
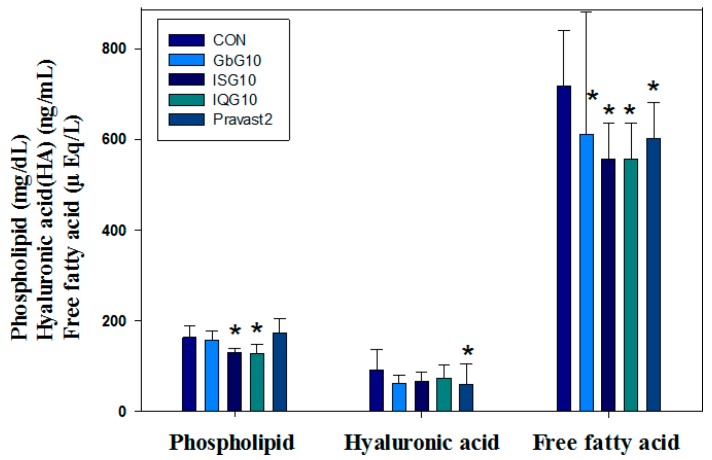
Antilipidemic effect on serum phospholipid, hyaluronic acid, and free fatty acid of insect GAG treated rats on HFD over one month. CON: control. GbG10: *G. bimaculatus* glycosaminoglycan 10 mg/kg. ISG10: *I. sinclairii* glycosaminoglycan 10 mg/kg. IQG10: *B. ignitus* glycosaminoglycan 10 mg/kg. Pravast2: Pravastain 2 mg/kg. (* *p* < 0.05).

In the sera of the IQG-treated groups (Table 2), phospholipid levels (mg/dL) were significantly lower (21%) than in the control after one month with dose-dependent changes in HFD-rats (CON, 163 ± 24.8; ISG10, 130.5 ± 9.4; IQG10, 128.6 ± 19.7, *p* < 0.05 vs. CON) (Figure 3). Also, the serum GPT (ALT) levels (IU/L) in IQG-treated groups were 39.1% lower than the control in HFD-rats (CON, 56.5 ± 25.2; GbG10, 48.9 ± 31.0; IQG10, 34.4 ± 9.8, *p* < 0.05) (Table 2). Total cholesterol levels (mg/dL) of IQG-treated rats were significantly lower (15.9%) than the control rats (CON, 108.6 ± 16.4; GbG10, 101.7 ± 9.8, *p* < 0.05; ISG10, 90.2 ± 11.3, *p* < 0.05; IQG10, 91.3 ± 10.6). In addition, a significantly decreased (90.5%) c-reactive protein (CRP) level (mg/L) was evident in the IQG-treated groups compared with the control (CON, 1.26 ± 0.40; GbG10, 0.22 ± 0.10; ISG10, 0.48 ± 0.20; IQG10, 0.12 ± 0.0, *p* < 0.05) (Table 2).

### 2.4. Anti-Oxidative Activity on Cellular Oxidative Damage

**Figure 4:**
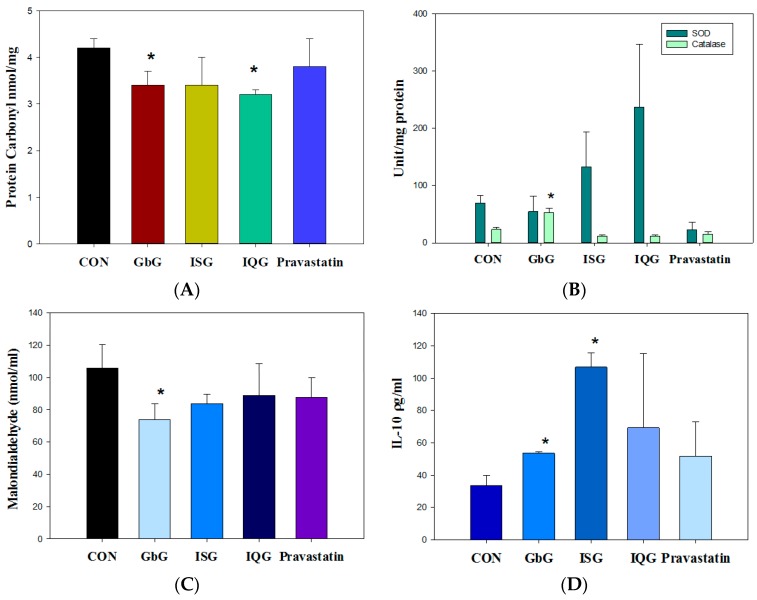
Anti-oxidative effect of some GAG on proteins (**A**) carbonyl, (**B**) Super oxide dismutase (SOD) or catalase content, and on lipids (**C**) malondialdehyde or (**D**) Interleukin-10. (* *p* < 0.05).

Protein oxidative stress was evaluated by measuring the protein carbonyl content in the blood. Catalase activity and carbonyl content were assayed as the biomarkers of protein oxidative damage in HFD rats. After 1 month, the carbonyl content level (nmol/mg protein) in the blood was decreased by each GAG treatment (CON, 4.2 ± 0.2; GbG10, 3.4 ± 0.3, GbG10 vs. CON, *p* < 0.05; ISG10, 3.4 ± 0.6; IQG10, 3.2 ± 0.1, IQG10 vs. CON, *p* < 0.05; Pravastatin, 3.8 ± 0). The protein carbonyl concentration was decreased at a ratio of 19.1%, 19.1%, and 23.8% in GbG10, ISG10, and IQG10, respectively (Figure 4A). Super oxide dismutase (SOD) is a free radical (super oxide) scavenger enzyme. SOD activities (nmol/mL) were increased compared to the control (CON, 69.64 ± 12.68; GbG10, 54.77 ± 26.72; ISG10, 133.13 ± 61.08, 191.1%; IQG10, 236.79 ± 110.27, 340.0%; Pravastatin, 22.91 ± 13.33) (Figure 4B). Catalase activity (nmol/mL) in hepatocytes after 1 month of each GAG treatment was as follows (CON, 23.41 ± 4.0; GbG10, 53.24 ± 7.47 (GbG10 vs. CON, *p* < 0.05); ISG10, 12.21 ± 1.24; IQG10, 11.46 ± 2.87; Pravastatin, 15.16 ± 4.56) (Figure 4B). The catalase activity in GbG-treated hepatocytes was increased by 227.4% (Figure 4B).

As a lipid oxidative damage marker in lipid oxidative stress states, malondialdehyde (MDA, nmol/mL) was assayed after 1 month of each GAG treatment (CON, 105.9 ± 14.6; GbG10, 73.8 ± 9.9, 30.32% decrease, GbG10 vs. CON, *p* < 0.05); ISG10, 83.8 ± 5.8; IQG10, 88.9 ± 19.6; Pravastatin, 87.6 ± 12.3). Each GAG and Pravastatin treatment decreased the lipid peroxidation in hepatocytes (Figure 4C).

### 2.5. Cytokine IL-1β and IL-10 Production

Serum IL-1beta levels were decreased and IL-10 levels were increased in each GAG treatment group. IL-1β (ρg/mL) in serum after one month was as follows: control, 840.0 ± 217.4; GbG10, 512.5 ± 132.6; ISG10, 706.25 ± 137.9 (ISG10 vs. CON, *p* < 0.05); IQG10, 535.0 ± 1.8; Pravastatin, 413.7 ± 38.9 (figure not shown). IL-10 activity (ρg/mL) after one month was as follows: CON: 33.6 ± 6.2, GbG10: 53.6 ± 0.9 (GbG10 vs. CON, *p* < 0.05), ISG10, 106.8 ± 8.8 (ISG10 vs. CON, *p* < 0.05): IQG10, 69.3 ± 46.0; Pravastatin, 51.8 ± 21.2 (Figure 4D). IQG10 and Pravastatin had no statistical differences compared with the control group.

### 2.6. Nitric Oxide Bioavailability and VEGF Levels in HMVEC

**Figure 5:**
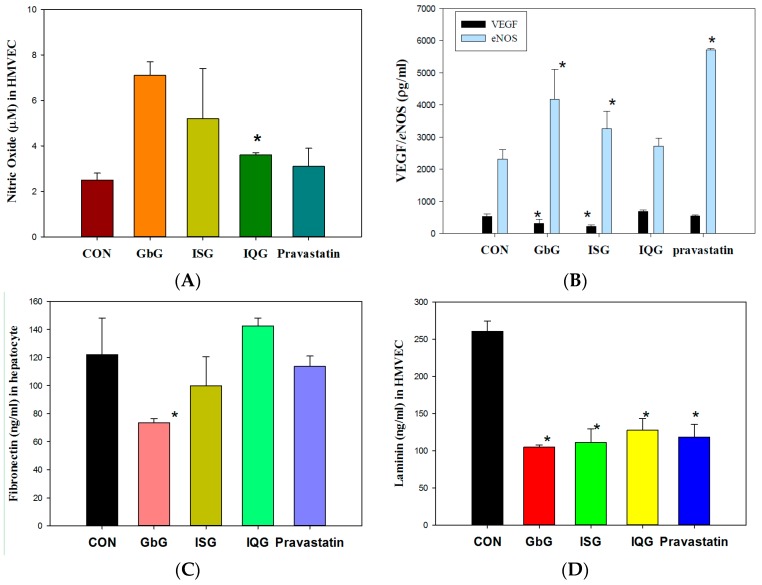
NO bioavailability of (**A**) nitric oxide, (**B**) Endothelial nitric oxide synthase, Endothelial nitric oxide synthase, *e*NOS and vascular endothelial growth factor, VEGF, (**C**) adhesion of fibronectin in hepatocytes, and (**D**) laminin on human microvascular endothelial cells, HMVEC-diabetic type cells. (* *p* < 0.05).

Nitric oxide (NO) was monitored as an endothelial vasorelaxation parameter. NO (μM) levels in human microvascular (cardiac) endothelial cells (D-HMVEC-C, diabetic type II) were increased by all treatments compared to the control, CON, 2.5 ± 0.3; GbG10, 7.1 ± 0.6; ISG10, 5.2 ± 2.2; IQG10, 3.6 ± 0.1 (144%, IQG10 vs. CON, *p* < 0.05); Pravastatin, 3.1 ± 0.8 (Figure 5A). Endothelial nitric oxide synthase, *e*NOS (ρg/mL) levels in the same cell type were increased by each GAG treatment (CON, 2308.3 ± 301.7; GbG10, 4170.6 ± 934.3 (180.6%, GbG10 vs. CON, *p* < 0.05); ISG10, 3255.0 ± 544.7 (141.0%, ISG10 vs. CON, *p* < 0.05); IQG10, 2715.0 ± 254.6; Pravastatin, 5715.0 ± 47.1 (Figure 5B). VEGF levels (ρg/mL) in HMVEC diabetic cells were changed by each GAG, but not significantly compared to the control except for the ISG10 group: CON, 527.2 ± 74.8; GbG10, 317.4 ± 122.3; ISG10, 220.9 ± 45.0, ISG10 vs. CON, *p* < 0.05; IQG10, 682.8 ± 49.0; Pravastatin, 553.0 ± 26.9 (Figure 5B).

### 2.7. Cytokine Laminin and Fibronectin Quantitation

The levels of fibronectin adhesive glycoproteins (ρg/mL) in hepatocytes were varied when compared to the control after 1 month (CON, 122.1 ± 26.0; GbG10, 73.6 ± 2.7, 60.2%, GbG10 vs. CON, *p* < 0.05; ISG10, 99.8 ± 20.9; IQG10, 142.5 ± 5.7; Pravastatin, 113.8 ± 7.2). In HMVEC diabetic cells, the fibronectin levels were substantially increased after day 2 of incubation (CON, 1058.8 ± 18.6; GbG10, 1093.8 ± 18.4; ISG10, 1181.0 ± 113.3; IQG10, 1327.6 ± 68.5; Pravastatin, 1064.8 ± 32.7) (Figure 5C). Laminin is a specialized extracellular matrix protein surrounding adipocytes. The level of laminin (ρg/mL) in HMVEC-d cells in the cells was significantly affected by the GAG treatments (CON, 260.4 ± 13.9; GbG10, 104.7 ± 3.0 (40.2%, GbG10 vs. CON, *p* < 0.05); ISG10, 111.2 ± 18.0 (ISG10 vs. CON, *p* < 0.05); IQG10, 127.6 ± 15.5; Pravastatin, 118.5 ± 16.9 (Figure 5D). However, the laminin level of HFD rat hepatocytes was not significant compared to the control: CON, 294.1 ± 7.3; GbG10, 307.7 ± 0.7; ISG10, 275.8 ± 15.8; IQG10, 286.1 ± 24.4; Pravastatin, 282.1 ± 18.2.

### 2.8. Fatty Acid Composition in Adipose Tissue

**Figure 6:**
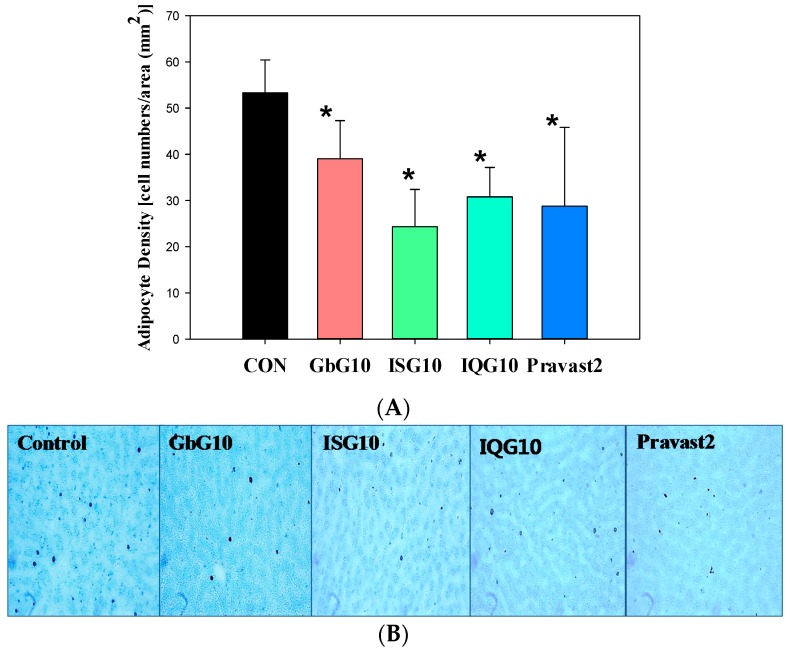
(**A**) Adipocyte number in rats treated with some GAG on HFD by toluidine blue O stain, (*n* = 10 per group) (* *p* < 0.05): significant difference vs. HFD group. HFD, high fat diet. The adipocyte cell density was counted from liver tissue toluidine blue stained depots. (**B**) Effect of IQG on morphology in the livers of rats fed HFD with IQG for one month. Representative toluidine blue O staining (fat cell depots) of liver tissue is shown at 400× magnification.

Adipocyte densities (cell/mm^2^) in tissues from treated rats were significantly reduced by GbG (39.06 ± 8.23), ISG (24.31 ± 8.07), IQG (36.75 ± 6.40), or Pravastatin (28.75 ± 17.08) compared to the control (53.31 ± 7.07) (Figure 6A). The adipocytes of rat liver tissues treated with each GAG in a HFD, as assessed by toluidine blue O staining, are shown in Figure 6B. The fatty acid profile as indicated by gas chromatography-mass spectrophotometer, showed a slight dose-dependent increase in the concentration of unsaturated fatty acids such as palmitic acid (C16:0), linoleic acid (C18:2), and oleic acid (C18:1) in the abdominal fat of male Wistar rats who received GbG for a 1 month period compared with the control group (Table 3). GAG-treated HFD rats displayed significant increases in the unsaturated fatty acid ratio in mono unsaturated fatty acids in epididymal fat, but decreases in saturated fatty acids (Table 4).

### 2.9. DNA Microarray 

Microarray analysis using a mouse 28 K cDNA clone array was performed to identify the gene expression profiles in liver tissue, and to provide information on potential markers for HFD diseases. Based on these results, GAG from the *Bombus ignitus* queen bee could prevent or treat fatty liver or hyperlipidemia in rats fed a HFD. Compared with the control, treatment with 10 mg/kg of this GAG resulted in up-regulation of 87 genes (test:control ratio >2.0). Up-regulated genes included the sema domain, immunoglobulin domain (Ig), short basic domain, secreted, (semaphorin) 3C (Sema3C), Fasin, fatty acid synthase (Fasn), expressed sequence tags moderately similar to Neuronal Protein 3.1 and similar to hypothetical protein MGC52110 (RGD1565095), 3-hydroxy-3-methylglutaryl-Coenzyme A reductase (Hmgcr), isopentenyl-diphosphate delta isomerase (Idil), and endothelin receptor type A (Ednra) (Table 5). Forty-seven genes were down-regulated (test:control ratio >0.5) including the uridine diphosphate (UDP) glycosyltransferase 2 family, polypeptide B (Ugt2b), insulin-like growth factor binding protein 1 (Igfbp1), serine dehydratase (Sds), ubiquitin D (Ubd), Myc (myelocytomatosis oncogene), and insulin receptor substrate 2 (Irs2) (Table 6).

These data suggest that Sema3c and Fasn were related to a reduction in fat, and were up-regulated by IQG treatment, indicating that these may be potential therapeutic markers for the successful treatment of HFD-related diseases.

### 2.10. Characterization of Used IQG and ISG

The compositions of the amino, acidic, and neutral monosaccharides of IQG were determined by GC-MS (Table 7). The primary amino monosaccharides detected in IQG were d-glucosaminic acid and *N*-acetyl-galactosamine, whereas in ISG they were d-glucosaminic acid and *N*-acetyl-d-gactosaminitol. The neutral monosugars of IQG were also small amounts (ng/mg) of α-glucose and mannitol, whereas in ISG, arabinose, β-glucose, and mannitol were found (Table 7). Monosaccharide composition of the used GbG was the same composition as that in reference 10.

## 3. Discussion

In this study, we demonstrated that IQG inhibits body weight gain, reduces adipose tissue weight, and repaired cellular oxidative damage in hepatocyte and blood cells. Furthermore, gene expression changes seen with IQG treatment involved RNA message changes for enzymes involved in lipid metabolism. That is, the anti-obesity effects of several insect GAGs, especially those produced by the *Bombus ignitus* queen bee or *Isaria sinclairii*, were revealed. The reduction of abdominal fat weight was observed (IQG < ISG < GbG < Pravastatin) as well as a less profound lowering of the blood pressure. In serum, the anti-inflammatory effect in HFD rats was seen by these GAGs, phospholipids, and CRPs (c-reactive protein) as the inflammatory parameter decreased. ISG or IQG GAGs reduced adipose tissue weight along with serum level normalization of ALP, ALT, LDH, and c-reactive protein, prolongation of the coagulation time to prevent blood aggregation in APPT (data not shown), and lipid accumulation in microvascular endothelial barriers that contribute to homeostasis in the circulatory system.

Activities indicative of potential benefits in the relief of HFD-related atherosclerosis and inflammatory, oxidative, or hyperglycemia conditions were also evident. Superoxide dismutase (SOD) is a free radical (super oxide) scavenging enzyme. Anti-obesity and lipid lowering effects were also related to antioxidant enzymes such as SOD [15]. In this experiment, these GAGs contributed to the increase of anti-oxidant activity and diminished cellular oxidative damage when anti-oxidative enzyme, SOD, or catalase activities were increased, whereas oxidative stress parameters, carbonyl content (protein oxidative damage), and malondialdehyde (MDA, lipid denaturant) in hepatocytes were diminished by the treatment of GbG, ISG, or IQG. In the present investigation, HFD rats treated with the IQG had lower MDA contents and higher SOD activity than those in the HFD group, indicating that this GAG can significantly suppress lipid peroxidation. IQG (feed for the *B. ignitus* queen is pollen known for having antioxidant materials) possesses potent antioxidant and free radical scavenging activities. These antioxidant activities could have contributed, at least partly, to the effect of IQG on suppressing lipid peroxidation and repairing protein damage (carbonyl content diminished).

As a metabolic change in response to a high fat diet, overexpression of *e*NOS prevents diet-induced obesity [16]. In this experiment, the NO level increased 144% and the *e*NOS level also increased 117% compared to the control in HMVEC cells by IQG10 treatment. Considering a pronounced anti-oxidative capacity of IQG, further experiments are needed to examine whether the increased NO bioavailability reflects augmented *e*NOS activity [17] or decreased degradation because of decreased superoxide levels.

As suggested in the report of Toita et al. (2016), the efficient delivery of therapeutic anti-inflammatory molecules, interleukin (IL)-10, to macrophages can dramatically improve the therapeutic efficacy of obesity treatments [18]. The treatment with IL-10-conjugated phosphatidylserine-containing liposomes with high affinity for macrophages exhibited significant anti-obesity and anti-inflammatory effects, such as reduced serum total cholesterol, adipocyte size, crown-like structures, pro-inflammatory cytokine secretion (IL-6 and tumor necrosis factor α) in adipose tissue, and liver injury in obese mice [18]. Therefore, we suspect that if these used GAGs could be conjugated with IL-10 in serum, as is our hope, then GAG-IL-10 could be causing the anti-obesity effect.

There were decreases in pro-inflammatory IL-1beta and increases in anti-inflammatory IL-10 at the same time in this experiment. The HFD groups increased the pro-inflammatory IL-1beta. Generally, no significant difference was observed regarding the anti-inflammatory cytokine IL-10 in the groups with the control and treatment. However, other studies showed that the effect of antioxidant compounds (e.g., Vitamin D) in other foods reduced pro-inflammatory molecules (IL-1beta) and increased anti-inflammatory markers, such as IL-10 [19].

Our study revealed that HFD induced the down-regulation of the IL-10 level and the used GAG treatment could sufficiently increase the expression of IL-10 to a greater extent than the control (ISG > IQG > GbG), such as the anti-obesity effect of HFD related reports [20]. However, in this experiment, IL-10 levels of the diluted (for assay) sample treated rat serum had a decreasing tendency with the passage of time. 

There are some reports on in vitro studies showing a strong decrease of fibronectin synthesis during adipocyte development whereas basement membrane molecules seem to increase during adipocyte differentiation [21]. In this experiment, during the inhibition of adipocyte development, the fibronectin level increased and the basement membrane molecule, laminin level was decreased by IQG or another GAG. Fibronectin maintains the balance between hemostasis and thrombosis [22], and its mechanobiology regulates tumorigenesis via VEGF [23]. In this study, the amount of cellular fibronectin in GbG-, ISG- or Pravastatin- treated rat hepatocytes was significantly decreased when compared to the control, whereas in D-HMVEC cells a slight increase was evident when compared to the control.

Meaningful gene expression profiles were evident in rats fed a HFD after treatment with IQG or ISG for one month. Class 3 semaphorins (Sema3C) secrete guidance proteins [24] and Fasn, which are involved in the adipose tissue transcriptional response of lipid metabolism [25], were upregulated. Ugt2b, UDP glycosyltransferase 2 family, polypeptide B [26], and Igfbp1 (insulin-like growth factor binding protein 1) [27] were down-regulated. UDP glycosyltransferase (UGT), mainly UDP glucuronosyltransferases, its subfamily UGT 2B enzymes related to xenobiotic-metabolizing enzymes and proteoglycan synthesis [28], and recapitulated dysregulation patterns of major UDP-glucuronosyltransferases (UGTs) were induced by HFD [29]. The presence of an additional N-linked glycan on the UGT2B7 variant enzyme resulted in a significant decrease in the formation of mycophenolic acid glucuronides [30]. Therefore, the prevalence of the HFD lifestyle nowadays, the combined treatment effect of HFD and the effect of these GAGs on disturbing UGTs was investigated for clinics by performing gene expression profiling. IGF I is a peptide hormone that is expressed in most tissues, to ameliorate myocardial growth and function post infarction [31]. However, in this experiment, Igfbp1 was down-regulated, but a high plasma level of Igfbp1, an insulin-like growth factor-binding protein-1, has recently been identified as a biomarker of amniotic fluid passage into the maternal circulation [32].

Squalene epoxidase, Sqle, is involved in the inhibition of lipogenesis and cholesterol synthesis, catalyzing the conversion of squalene to 2,3-oxidosqualene [33]. Presently, the insect GAGs up-regulated the production of Sqle expression, which can block cholesterol synthesis as hypocholestrolemic agents [34,35]. To summarize the gene expression profiling, Fasn, adipose tissue transcriptional response of the lipid metabolism gene was up-regulated and the UDP glycosyltransferase 2 family, polypeptide B (Ugt2b) was down-regulated in GbG, ISG, or IQG-treated rats.

GAGs are complex linear polysaccharides expressed in intracellular compartments at the cell surface, and in the extracellular environment where they interact with various molecules to regulate many cellular processes implicated in health and disease [36]. For example, the loss of GAG receptor binding after mosquito cell passage reduces Chikungunya virus infectivity [37]. More knowledge of the structure of GAGs would be advantageous. These studies are challenging due to the lability of the constituent sulfate groups, very diverse modifications, and the epimerization of the uronic acids [38]. The molecular structure of IQG is supposed to be composed of *N*-acetylhexosamine and hexosamine, that is, *N*-acetyl-galactosamine and d-glucosaminic acid by the GC-MS analysis results. Also, the molecular mass of the ISG moieties were d-glucosaminic acid and *N*-acetyl-d-gactosaminitol in this experiment. The ISG co-incubated heparin-lyase pattern was matched to heparin disaccharides according to a previous report [39]. Perhaps, the functional superiority of the used insect GAG does not originate from structural complication (not a more complicated form from animal GAG) but from insect feed diversity such as pollen (IQG) or *I. sinclarii* fungi derived from silkworms (ISG). Therefore, GAGs derived from insects could also be a safer replacement than heparin and other GAGs from mammalian sources, as there is a high possibility of transmission of infectious agents such as foot-mouth disease from sources like pigs [10].

## 4. Materials and Methods

### 4.1. Preparation of Insect Glycosaminoglycan

*Bombus ignitus* queens were reared and *I. sinclairii* was cultivated with forming fruiting bodies in the Department of Agricultural Biology, National Academy of Agricultural Science, Wanju, Korea. *G. bimaculatus* were reared at a cricket farm located in Jungsun, Korea. Theses insects were freeze-dried at the same institute. All reagents were supplied from Sigma Aldrich (St. Louis, MO, USA).

Dried insect material (1 kg) was soaked and extracted three times with ethanol by ultrasonification (Branson, Colorado, MI, USA) for 30 min. The residues separated from the alcohol extracts were defatted twice with two volumes of acetone. Approximately 200 g of dried, defatted, and pulverized powder was suspended in 2 L of 0.05 M sodium carbonate buffer (pH 9). The suspension was incubated for 48 h at 60 °C after adding 28 mL (1.4%) Alcalase. The digestion mixture was cooled to 4 °C, and trichloroacetic acid was added to a final concentration of 5%. The sample was mixed, allowed to stand for 1 h, and centrifuged for 30 min at 8000× *g* (Hanil Science Industrial, Incheon, South Korea). Three volumes of 5% potassium acetate in ethanol were added to one volume of supernatant. After mixing, the suspension was stored overnight at 4 °C and centrifuged. The precipitate amounting to 20 g was dissolved in 40 mL of 0.2 M NaCl and centrifuged. Cetylpyridinium chloride (5%) was added to 0.2 times the volume of the supernatant, and the precipitate was collected by centrifugation. The precipitate was dissolved in 20 mL of 2.5 M NaCl. Five volumes of ethanol were added, and the precipitate was separated by centrifugation. The precipitate was dissolved in water and dialyzed against 100 volumes of water [2], and the dialyzed crude GAG was freeze-dried to obtain about 1.7 g of GbG, 1.56 g of ISG, 4.89 g of IQG as a powder. Crude GAG was loaded onto a DEAE Sephadex A-25 gel chromatography column (40 cm × 1.2 cm) equilibrated with 50 mM phosphate buffer (pH 7.4). The fractions were eluted using a linear sodium chloride gradient from 0 to 2.5 M NaCl in phosphate buffer at a flow rate of 20 mL/h, and the dialyzed glycan was freeze-dried to obtain GAG.

### 4.2. Animal Handling

**Figure 7:**
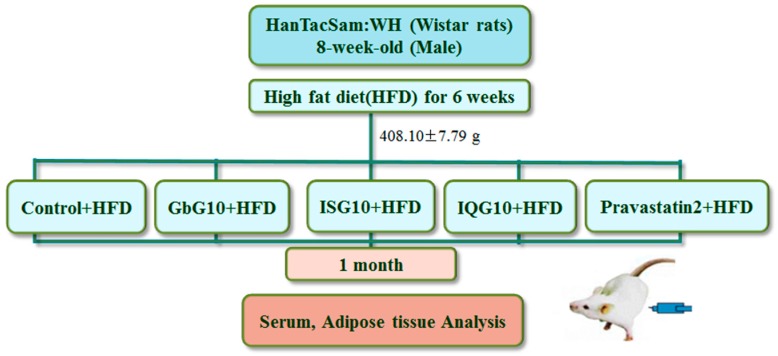
Animal experimental design. GbG: *G. bimaculatus* glycosaminoglycan; ISG: *I. sinclairii* glycosaminoglycan; IQG: *B. ignitus* glycosaminoglycan; Pravast2: Pravastain 2 mg/kg.

Male, 8-week-old, Han Tac Sam-WH (Wistar) rats were purchased from Samtako Co., Ltd. (Osan, Korea). All procedures were performed in accordance with the NIH Guidelines for Care and Use of Laboratory Animals. All experiments were approved by the Laboratory Animals’ Ethical Committee of the Rural Development Administration, National Academy of Agricultural Science (NAAS 1501), and followed national guidelines for the care and use of animals. The rats were housed individually and were acclimated for 6 weeks under normal husbandry conditions (23 ± 2 °C, 55% ± 10% humidity, and 12 h light/dark cycle) and fed a HFD (D12492, 60% fat; Research Diet Inc., New Brunswick, NJ, USA). The diet and water were available ad libitum. The rats were segregated into five treatment groups of 10 rats each with a similar weight distribution in the groups (408.10 ± 7.79 g). The suspensions in phosphate buffered saline (PBS) were given intraperitoneally each day. The groups were control (PBS only; CON), 10 mg/kg GbG (GbG10), 10 mg/kg ISG (ISG10), 10 mg/kg IQG (IQG10), and 2 mg/kg Pravastatin (Pravastain2) (CJ Healthcare Co., Seoul, Korea) (Figure 7).The HFD diet and sample treatment was maintained for one month.

#### Declaration of Ethics Approval and Consent to Participate

Studies involving animals were approved by the Laboratory Animals’ Ethical Committee of the National Academy of Agricultural Science, RDA, South Korea (NAAS 1501). Manuscripts reporting studies did not contain human participants, human data, or human tissues.

### 4.3. Blood Pressure

Blood pressure was measured by the indirect tail-cuff method on a weekly basis as described previously [11] using a model MK-V100 blood monitoring system (Muromachi Kikai Co., Tokyo, Japan). Measurements were performed one week before treatment and after 4 weeks of treatments.

### 4.4. Organ and Adipose Tissue Weights

Absolute and relative weights (organ-to-body ratio) were measured for the adrenal glands, kidneys, heart, liver, lung, spleen, stomach, pancreas, thymus, and ovaries. Abdominal and epididymal fat-to-body weight ratios were also determined. The measurements were made after sacrifice at the end of the one-month HFD.

### 4.5. Blood Sampling and Serum Assay

After the 1-month HFD, blood (~5 mL) was collected from the posterior vena cava under light CO_2_ inhalation and used for serum chemistry measurements. The parameters examined included phospholipid, hyaluronic acid, free fatty acid (FFA), albumin, alkaline phosphatase (ALP), creatinine phosphokinase (CK), glutamic oxaloacetic transaminase (GOT), glutamic pyruvic transaminase (GPT), lactic dehydrogenase (LDH), glucose, total cholesterol, high-density lipoprotein cholesterol (HDL-C), low-density lipoprotein cholesterol (LDL-C), triglyceride, blood urea nitrogen (BUN), creatinine, uric acid, total protein, and c-reactive protein (CRP). All parameters were evaluated using a model 7060 automatic clinical analyzer (Hitachi, Tokyo, Japan).

### 4.6. Liver Homogenate Preparation for Oxidative Enzyme Detection

Liver tissues were homogenized on ice in 10 volumes of lysis buffer (PRO-PREP^TM^ protein extraction solution; *i*NtRON, Busan, Korea). The supernatant of the liver homogenate after centrifugation (800× *g*, 10 min) was assayed for superoxide dismutase activity or catalase activity according to the assay manual (Cayman ELISA kit, Cayman Chemical Co., Ann Arbor, MI, USA).

### 4.7. Oxidative Protein Damage 

Liver homogenate supernatants and blood, obtained following centrifugation, were used for the determination of carbonyl content and catalase (CAT) activity. Carbonyl content was determined with an enzyme-linked immunoassay according to the manufacturer’s protocol for the OxiSelect™ protein carbonyl ELISA kit (Cell Biolabs, Inc., San Diego, CA, USA). CAT activity (U/mg protein) was measured based on the CAT-mediated decomposition of H_2_O_2_ [40]. Potassium phosphate buffer (50 mM, pH 7.0, 0.9 mL) was added to 0.1 mL of the sample followed by a 1 mL, 30 mM H_2_O_2_ solution. The decrease in the absorbance at 240 nm was measured for 90 s.

### 4.8. Oxidative Lipid Damage 

To determine the oxidative lipid damage in rat hepatocytes, malondialdehyde (MDA) levels were measured with a lipid peroxidation assay using the color method involving thiobarbituric acid reactive substances (TBARS) at 535 nm [41]. The liver homogenate (0.5 mL) obtained as described above was incubated along with 1 mL 7% sodium dodecyl sulfate (SDS) for 30 min at 37 °C before being mixed with 2 mL of 0.67% TBA (1:1 with acetic acid) and added to tubes. The tubes were mixed, placed in boiling water (100 °C) for 50 min, and then mixed with 5 mL butanol. 1,1,3,3-tetraethoxypropane was used as a standard.

### 4.9. Cytokine IL-1β and IL-10 Assay

The IL-1β or IL-10 levels in rat serum were measured using commercial ELISA kits (Quantikine; R&D Systems, Inc., Minneapolis, MN, USA) according to the manufacturer’s instructions.

### 4.10. NO Assay and Growth Factor in Microvascular Endothelial Cells 

VEGF production was measured in human microvascular (cardiac) endothelial cells (D-HMVECs) obtained from type 2 diabetics (Clonetics™, diabetic type II, Lonza CC-2928, D-HMVEC-C diabetic type II, Cambrex, Walkersville, MD, USA). Cells were grown in an endothelial cell basal medium (EBM)-2 with EGM-2 singlequots (Cambrex) at 37 °C in an atmosphere containing 5% CO_2_. Cells pretreated with 0.2 mg/mL of each individual GAG or Pravastatin were incubated prior to the determination of NO (Cayman Chemical, Ann Arbor, MI, USA), endothelial nitric oxide synthase (*e*NOS), and VEGF (Quantikine, R&D Systems, Inc., Minneapolis, MN, USA) as previously reported [5], according to manufacturers’ instructions.

### 4.11. Cytokine Laminin and Fibronectin Assay

The laminin and fibronectin levels in rat serum were measured using Quantimatrix™ human fibronectin or laminin ELISA kits (Millipore, Billerica, MA, USA) in HMVEC cells or the above mentioned hepatocytes, according to the manufacturer’s instructions.

### 4.12. DNA Microarray

After histopathological analysis, microarray hybridization was performed on liver samples as described previously [10,42]. Total RNA was isolated from the liver using a RNeasy Midi Kit (Qiagen, Valencia, CA, USA). A FairPlay™ microarray labeling kit (Stratagene, La Jolla, CA, USA) was employed according to the manufacturer’s instructions. The labeled DNA was loaded onto a microarray chip. A hybridization chamber was assembled with that chip and submerged in a water bath overnight at 60 °C. The microarray chip was washed in a wash buffer I (2× SSC, 0.1% SDS) for 15 min, in wash buffer II (1× SSC) for 5 min, and in wash buffer III (0.2× SSC) for 15 min. Each slide containing the chip was dried by centrifuging at 500× *g* for 15 min and scanned with a BMS Array Scanner, connected to an Applied precision Array WoRx eBiochip Reader (BioRad, Dallas, TX, USA) using the Cy3 and Cy5 channels [43].

### 4.13. Analysis of Fatty Acid Composition in Rat Adipose Tissue 

Adipocyte density (cells/mm^2^) was determined in treated and control tissue by toluidine blue O staining (original magnification, ×400).

For epididymidal and abdominal fat analysis, the concentrations of free fatty acids and fatty acid composition were determined for 29 fatty acids in adipose tissues by gas chromatography-mass spectrometry (GC-MS). Adipose or epididymidal tissues (0.1 g) were collected and fatty acid was extracted by an overnight treatment with chloroform and methanol (2:1). The solution was filtered and the liquid was removed under a flow of nitrogen gas. The remaining lipids were saponified by alkaline hydrolysis of phospholipids at 100 °C with 0.5 N methanolic sodium hydroxide and methylated at 100 °C with 14% boron trifluoride for 15 min. The top layer was transferred in petroleum ether and analyzed by GC-MS using a model 6890GC 5973N mass detector operating in electroscopy ionization mode (Agilent, Santa Clara, CA, USA) equipped with a HP-5 capillary column (Agilent). The inlet temperature was 250 °C and the MS transfer line was kept constant at 230 °C. The oven temperature was held at 180 °C for 20 min, then increased at a rate of 10 °C/min and was held at 230 °C for 10 min. Quantification was achieved using a mixed 37-fatty acid standard at 10 ng/mL Sigma Aldrich (Sigma-Aldrich, St. Louis, MO, USA). Linoleic acid (C18:2n6) (Sigma-Aldrich) was used as an internal standard.

### 4.14. Statistical Analyses

The mean and standard error of all parameters were determined for each group using ANOVA. Student’s *t*-test was carried out to determine significant differences between the control and treated groups. A *p*-value < 0.05 was considered significant. 

## 5. Conclusions

The collected findings suggest that GAG could be a natural anti-lipidemic agent, with comparable activities to more expensive commercial drugs such as heparin/heparan sulfate. Insect GAG could be adapted as a functional food, similar to commercial *N*-acetyl glucosamine. New glycosaminoglycan purified from the queen of *B. ignitus* as an insect source, IQG, displayed anti-lipidemic effects in serum biochemical parameter levels and a change in the fatty acid composition of adipose tissues, showing an increased ratio of conjugated unsaturated fatty acids. Thus, this compound might have potential for use as an anti-atherosclerotic agent.

## Figures and Tables

**Table 1:** Blood pressure values of insect glycosaminoglycan in rats on a high fat diet over a one month period.

Parameter (Unit)	Heart Rate (beat/min)	Systolic BP (mmHg)	Median BP (mmHg)	Diastolic BP (mmHg)
CON	404.93 ± 24.42	155.18 ± 23.84	107.34 ± 21.25	79.88 ± 21.02
GbG10	398.30 ± 41.31	144.80 ± 20.58 ***	91.70 ± 31.86	76.47 ± 24.96
ISG10	391.62 ± 37.76	138.84 ± 30.48	87.14 ± 33.94 *	65.48 ± 30.03 *
IQG10	403.23 ± 17.23	140.04 ± 18.41	90.23 ± 21.79 *	61.87 ± 22.20 *
Pravastatin2	399.28 ± 19.94	142.91 ± 36.89	92.28 ± 33.61	70.67 ± 29.84

Marginal statistically significant differences in blood pressure and heart rate were observed between the 10 mg/kg GbG-, ISG-, and IQG-treated groups compared to the control (* *p* < 0.05, *** *p* < 0.001). Each value represents mean ± S.D. BP: blood pressure.

**Table 2:** Serological finding of insect glycosaminoglycan of high fat diet rats treated with insect glycosaminoglycan over a one month period.

Parameter	Unit	CON	GbG10	ISG10	IQG10	Provast2
Albumin	g/dL	4.42 ± 0.16	3.82 ± 0.26 *	3.91 ± 0.15 *	3.5 ± 0.21 *	4.27 ± 0.12
ALP	U/L	92.89 ± 38.45	118.3 ± 20.79	56.88 ± 9.91 *	95.2 ± 27.52	89.0 ± 20.65
AST(SGOT)	U/L	134.25 ± 48.49	145.8 ± 44.72	102.88 ± 17.63	123.56 ± 40.67	148.7 ± 45.29
ALT(SGPT)	U/L	56.56 ± 25.24	48.9 ± 31.08 *	37.5 ± 6.65	34.44 ± 9.85 *	55.9 ± 20.01
CK	U/L	862.0 ± 525.93	933.7 ± 279.11	650.88 ± 371.49	749.22 ± 623.93	771.6 ± 367.47
LDH	U/L	1127.8 ± 97.75	1200.0 ± 0 *	1102.75 ± 160.04	1058.1 ± 299.22	1161.6 ± 121.43
Glucose	mg/dL	257.67 ± 157.3	211.3 ± 57.17	328 ± 94.73	198.5 ± 90.21	252.5 ± 93.76
T.Chol	mg/dL	108.67 ± 16.48	101.7 ± 9.89 *	90.25 ± 11.32 *	91.3 ± 10.6	121.9 ± 23.38
Triglyceride	mg/dL	128.22 ± 54.88	147.13 ± 31.1	105.88 ± 18.6	98.9 ± 28.96	134.2 ± 66.11
LDL Chol	mg/dL	22.0 ± 5.73	23.2 ± 5.16	15.33 ± 4.44	19.6 ± 5.23	25.9 ± 7.82
HDL Chol	mg/dL	84.44 ± 11.17	73.4 ± 7.47 *	76.63 ± 9.52	69.5 ± 10.92 *	95.8 ± 15.18
Creatinine	mg/dL	0.54 ± 0.08	0.53 ± 0.04	0.63 ± 0.06 *	0.55 ± 0.08	0.57 ± 0.06
BUN	mg/dL	18.34 ± 3.2	23.76 ± 1.79 *	17.35 ± 2.03	16.83 ± 3.01	19.84 ± 3.43
Uric acid	mg/dL	5.01 ± 2.97	3.8 ± 0.94	8.65 ± 2.36 *	5.89 ± 2.22	5.17 ± 1.67
Protein, total	g/dL	6.91 ± 0.27	6.91 ± 0.44	6.83 ± 0.09	7.01 ± 0.29	6.89 ± 0.19
CRP(HS)	mg/L	1.26 ± 0.44	0.22 ± 0.17 *	0.48 ± 0.27 *	0.12 ± 0.04 *	0.86 ± 0.32

CON: control. GbG10: G. bimaculatus glycosaminoglycan 10 mg/kg. ISG10: I. sinclairii glycosaminoglycan 10 mg/kg. IQG10: B. ignitus glycosaminoglycan 10 mg/kg. Pravast2: Pravastain 2. ALP: alkaline phosphatase; AST(GOT): glutamate oxaloacetate transaminase; ALT(GPT): glutamate pyruvate transaminase; CK: creatinine phosphokinase; LDH, lactate dehydrogenase; BUN, blood urea nitrogen; T. Chol: total cholesterol; HDL Chol: high cholesterol; LDL Chol: low cholesterol; CRP: c-reactive protein. Each value represents mean ± S.D., statistically significant from the control (* *p* < 0.05).

**Table 3:** Fatty acid composition of abdominal fat of high fat diet rats treated with insect glycosaminoglycan over a one month period.

Comp. of Abdominal Fat Tissue (%)	Control	GbG10	ISG10	IQG10	Pravastatin
Lauric acid (C12:0)	2.90 ± 2.05	1.97 ± 1.39	4.94 ± 0.22	3.56 ± 0.72	4.90 ± 0.33
Myristoleic acid (C14:1)	N.D.	15.25 ± 5.30	14.95 ± 2.65	24.28 ± 13.12	N.D.
Palmitoleic acid (C16:1)	15.18 ± 10.73	18.31 ± 12.95	2.31 ± 1.63	20.55 ± 7.84	26.71 ± 14.09
Palmitic acid (C16:0)	4.01 ± 1.81	5.73 ± 2.61	8.54 ± 5.92	18.00 ± 11.75	16.48 ± 10.91
Linoleic acid (C18:2)	10.44 ± 7.39	18.38 ± 4.10	10.76 ± 2.15	13.53 ± 4.29	11.07 ± 2.94
Oleic acid (C18:1)	5.06 ± 3.28	16.24 ± 2.35	28.05 ± 7.86	13.46 ± 1.87	18.76 ± 4.64
Stearic acid (C18:0)	43.19 ± 12.01	18.83 ± 1.43	25.89 ± 11.00	3.01 ± 0.20	11.05 ± 2.24
Arachidonic acid (C20:4)	5.44 ± 1.48	2.18 ± 0.35	1.70 ± 0.11	1.78 ± 0.69	4.37 ± 0.70
Eicosapentaenoic acid (C20:5)	1.14 ± 0.34	1.84 ± 0.21	1.55 ± 0.16	0.88 ± 0.22	2.80 ± 0.49
Eicosatrienoic acid (C20:3)	6.00 ± 2.76	0.27 ± 0.14	0.22 ± 0.12	0.18 ± 0.01	0.59 ± 0.18
Eicosadienoic acid (C20:2)	1.56 ± 0.23	0.48 ± 0.13	0.42 ± 0.15	0.22 ± 0.01	0.75 ± 0.24
Eicosenoic acid (C20:1)	3.17 ± 1.34	0.44 ± 0.11	0.51 ± 0.05	0.28 ± 0.01	1.65 ± 0.73
Eicosanoic acid (C20:0)	1.60 ± 0.05	0.02 ± 0.00	0.11 ± 0.01	0.26 ± 0.10	0.66 ± 0.29
Docosahexaenoic acid (C22:6)	0.33 ± 0.00	0.08 ± 0.05	0.04 ± 0.01	0.02 ± 0.00	0.21 ± 0.05
Saturated fatty acid	51.69	26.54	39.48	42.33	33.09
Unsaturated fatty acid	48.31	73.46 *	60.52	57.67	66.91
Mono unsaturated fatty acid	23.41	50.24	45.82	48.35	47.12
Poly unsaturated fatty acid	24.91	15.18	14.70	16.61	19.79

Each value represents mean ± SD. Asterisk marks (*) mean significant differences compared with the control (PBS) group (*p* < 0.05). N.D.: not detected.

**Table 4:** Fatty acid composition of epididymal fat of high fat diet rats treated with insect glycosaminoglycan over a one month period.

Comp. of Epididymial Fat Tissue (%)	Control	GbG10	ISG10	IQG10	Pravastatin
Lauric acid (C12:0)	2.65 ± 4.35	3.26 ± 4.53	7.83 ± 4.70	4.56 ± 4.22	1.83 ± 0.45
Myristoleic acid (C14:1)	N.D.	0.96 ± 1.36	5.84 ± 8.24	N.D.	7.43 ± 8.19
Palmitoleic acid (C16:1)	10.26 ± 6.58	22.12 ± 14.15	8.67 ± 8.97	55.99 ± 11.37	25.41 ± 22.01
Palmitic acid (C16:0)	38.67 ± 3.32	45.84 ± 8.39	24.96 ± 9.53	7.10 ± 4.07	19.84 ± 15.49
Linoleic acid (C18:2)	4.53 ± 3.60	14.12 ± 9.35	7.75 ± 4.06	5.39 ± 6.62	3.04 ± 2.64
Oleic acid (C18:1)	10.25 ± 5.24	2.99 ± 1.16	0.53 ± 0.67	2.04 ± 1.73	17.52 ± 13.91
Stearic acid (C18:0)	6.39 ± 1.85	0.10 ± 0.14	4.19 ± 5.93	4.75 ± 6.65	6.10 ± 5.86
Arachidonic acid (C20:4)	10.88 ± 6.60	3.69 ± 4.89	18.76 ± 8.57	7.96 ± 1.33	13.81 ± 9.26
Eicosapentaenoic acid (C20:5)	11.93 ± 7.90	3.42 ± 4.68	16.95 ± 6.85	6.39 ± 1.11	1.36 ± 1.19
Eicosatrienoic acid (C20:3)	2.43 ± 2.95	1.03 ± 1.28	1.71 ± 1.71	1.92 ± 2.32	0.74 ± 0.41
Eicosadienoic acid (C20:2)	1.14 ± 0.52	1.38 ± 1.73	1.26 ± 1.25	1.44 ± 0.51	1.55 ± 1.17
Eicosenoic acid (C20:1)	0.31 ± 0.20	0.85 ± 1.12	0.34 ± 0.41	0.76 ± 0.53	0.21 ± 0.21
Eicosanoic acid (C20:0)	0.37 ± 0.30	0.07 ± 0.07	0.78 ± 0.40	1.12 ± 0.74	1.04 ± 1.55
Docosahexaenoic acid (C22:6)	0.18 ± 0.08	0.17 ± 0.04	0.43 ± 0.03	0.57 ± 0.86	0.12 ± 0.06
Saturated fatty acid	48.08	49.28	37.76	17.53	28.82
Unsaturated fatty acid	51.92	50.72	62.24	82.47	71.18
Mono unsaturated fatty acid	20.83	26.92	15.38	58.80 *	50.58 *
Poly unsaturated fatty acid	31.09	23.80	46.87 *	23.68	20.61

Each value represents mean ± SD. Asterisk marks (*) mean significant differences compared with the control (PBS) group (*p* < 0.05). N.D.: not detected.

**Table 5:** Up-regulated genes differentially expressed in the liver tissue of high fat diet rats treated with insect glycosaminoglycan over a one month period.

Description		Gene Symbol	IQG	GbG	ISG
1	sema domain, immunoglobulin domain (Ig), secreted, (semaphorin) 3C	*Sema3c*	2.60	0.93	1.03
2	fatty acid synthase	*Fasn*	2.45	1.81	2.27
3	similar to hypothetical protein MGC52110	*RGD1565095*	2.36	0.85	1.18
4	3-hydroxy-3-methylglutaryl-Coenzyme A reductase	*Hmgcr*	2.26	1.27	1.09
5	similar to hypothetical protein MGC47256	*RGD1308694*	2.21	1.04	1.04
6	isopentenyl-diphosphate delta isomerase 1	*Idi1*	2.12	1.64	1.28
7	endothelin receptor type A	*Ednra*	2.02	0.97	1.03
8	squalene epoxidase	*Sqle*	1.99	2.29	1.11
9	pleckstrin homology-like domain, family A, member 3	*Phlda3*	1.98	1.37	1.06
10	reticulon 4	*Rtn4*	1.98	0.90	1.09
11	PHD finger protein 11	*Phf11*	1.88	0.98	1.00
12	apolipoprotein L 9a	*Apol9a*	1.88	1.31	0.92
13	malic enzyme 1, NADP(+)-dependent, cytosolic	*Me1*	1.87	1.17	1.17
14	similar to zinc finger protein 146	*LOC687516*	1.86	1.06	1.06
15	myeloid/lymphoid or mixed-lineage leukemia, translocated to, 3	*Mllt3*	1.83	0.85	0.89
16	Eph receptor A2	*Epha2*	1.79	1.17	1.17
17	sterol-C4-methyl oxidase-like	*Sc4mol*	1.77	1.95	1.82
18	lanosterol synthase (2,3-oxidosqualene-lanosterol cyclase)	*Lss*	1.73	1.41	1.52
19	solute carrier family 2, (facilitated glucose transporter) member 8	*Slc2a8*	1.72	0.93	0.95
20	cytochrome P450, family 4, subfamily a, polypeptide 8	*Cyp4a8*	1.72	1.09	1.26
21	ephrin A1	*Efna1*	1.72	1.25	0.92
22	cysteine and glycine-rich protein 1	*Csrp1*	1.70	1.76	1.42
23	claudin 1	*Cldn1*	1.70	1.50	0.90
24	aspartoacylase	*Asp* *A*	1.69	1.12	0.92
25	7-dehydrocholesterol reductase	*Dhcr7*	1.69	1.74	1.45
26	hydroxyacid oxidase 2 (long chain)	*Hao2*	1.69	1.41	1.29
27	Inhibin beta-A	*Inhba*	1.68	1.47	0.89
28	YKT6 v-SNARE homolog (S. cerevisiae)	*Ykt6*	1.68	1.11	1.04
29	WD repeat domain 89	*Wdr89*	1.68	1.05	1.00
30	farnesyl diphosphate synthase	*Fdps*	1.65	1.11	1.42

Each DNA microarray ratio of IQG, ISG, or GbG is the pair mean ratio (test/control) of *B. ignitus glycosaminoglycan* 10 mg/kg, *I. sinclairii* glycosaminoglycan 10 mg/kg, *G. bimaculatus* glycosaminoglycan 10 mg/kg.

**Table 6:** Down-regulated genes differentially expressed in the liver tissue of high fat diet rats treated with insect glycosaminoglycan over a one month period.

Description		Gene Symbol	IQG	ISG	GbG
1	UDP glycosyltransferase 2 family, polypeptide B	*Ugt2b*	0.10	1.04	0.92
2	insulin-like growth factor binding protein 1	*Igfbp1*	0.15	0.72	1.09
3	serine dehydratase	*Sds*	0.23	1.10	0.44
4	ubiquitin D	*Ubd*	0.28	3.21	0.84
5	myelocytomatosis oncogene	*Myc*	0.28	0.94	1.16
6	insulin receptor substrate 2	*Irs2*	0.35	1.02	1.15
7	growth arrest and DNA-damage-inducible, gamma	*Gadd45g*	0.39	0.99	2.80
8	dual specificity phosphatase 1	*Dusp1*	0.40	0.96	1.06
9	early growth response 1	*Egr1*	0.41	1.00	1.25
10	similar to Protein C8orf4 (Thyroid cancer protein 1) (TC-1)	*LOC684871*	0.41	0.77	0.93
11	cytokine inducible SH2-containing protein	*Cish*	0.42	1.41	1.59
12	similar to interferon regulatory factor 2 binding protein 2	*LOC679357*	0.43	0.71	1.09
13	solute carrier family 38, member 2	*Slc38a2*	0.44	1.08	1.27
14	polymerase (DNA directed), gamma 2, accessory subunit	*Polg2*	0.45	0.90	0.73
15	glutamic-oxaloacetic transaminase 1, soluble (aspartate aminotransferase 1)	*Got1*	0.46	0.87	0.67
16	hematopoietically expressed homeobox	*Hhex*	0.47	1.11	1.42
17	nuclear receptor subfamily 1, group D, member 2	*Nr1d2*	0.48	1.50	0.75
18	coiled-coil domain containing 52	*Ccdc52*	0.49	1.02	1.02
19	kininogen 1 /kininogen 1-like 1	*Kng1/Kng1l1*	0.50	1.14	1.82
20	WD repeat and SOCS box-containing 1	*Wsb1*	0.50	0.90	1.04
21	frizzled homolog 1 (Drosophila)	*Fzd1*	0.50	1.02	1.09
22	Kruppel-like factor 6	*Klf6*	0.50	1.00	0.79
23	fibroblast growth factor 21	*Fgf21*	0.51	0.82	1.17
24	kruppel-like factor 15	*Klf15*	0.51	1.00	0.90
25	similar to DNA segment, Chr 16, ERATO Doi 472, expressed	*RGD1563888*	0.52	0.79	0.94
26	nuclear factor of kappa light polypeptide gene enhancer in B-cells inhibitor, zeta	*Nfkbiz*	0.52	0.99	1.08
27	interleukin 6 receptor, alpha	*I16ra*	0.52	0.95	0.92
28	dual specificity phosphatase 1	*Duspl*	0.53	0.92	0.95
29	Htra serine peptidase 3	*Htra*	0.53	0.97	0.94
30	MCG147639-like	*LOC100363606*	0.53	0.87	0.79

Each DNA microarray ratio of IQG, ISG, or GbG is the pair mean ratio (test/control) of of *B. ignitus* glycosaminoglycan 10 mg/kg, *I. sinclairii* glycosaminoglycan 10 mg/kg, *G. bimaculatus* glycosaminoglycan 10 mg/kg.

**Table 7:** Monosaccharide composition of purified ISG and IQG.

Acidic and Amino Sugar	ISG	IQG	Monosugar	ISG	IQG
	(μg/mL)			(ng/mL)	
d-Glucuronic Acid	82.91	40.66	Arabinose	273.86	3.08
Glucosamine HCl	3.33	25.57	Rhamnose	0.0	2.65
Galactosamine HCl	25.64	69.41	Ribose	7.41	0.0
*N*-Acetyl-Glucosamine	42.92	219.84	Xylose	4.79	0.0
d-Glucosaminic Acid	530.64	389.91	Xylitol	0.0	0.0
d-Galactosamic Acid	49.42	47.93	Mannose	6.44	0.0
*N*-Acetyl-d-Galactosaminitol	159.15	103.94	Frutose	0.66	0.0
			Galactose	17.52	0.0
d-Glucosamine-6-phosphate	15.56	35.09	α-glucose	78.46	14.73
Total Sum	909.56	932.35	Mannitol	162.75	12.78
			β-glucose	169.46	1.95

## Abbreviations

GbG10: *Gryllus bimaculatus* (a type of cricket) glycosaminoglycan 10 mg/kg; ISG10: *Isaria sinclairii* glycosaminoglycan 10 mg/kg; IQG10: queen of *Bombus ignitus* (a type of bumblebee) glycosaminoglycan 10 mg/kg; Pravast2: pravastatin 2 mg/kg; ALP: alkaline phosphatase; AST(GOT), glutamate oxaloacetate transaminase; ALT(GPT), glutamate pyruvate transaminase; BUN, blood urea nitrogen; CK: creatinine phosphokinase; CRP: c-reactive protein; LDH, lactate dehydrogenase; T. Chol: total cholesterol; HDL Chol: high cholesterol; LDL Chol: low cholesterol.
